# A Comparison of Ultrasound Guided Curettage With and Without Uterine Artery Embolization on Controlling Intraoperative Blood Loss for a Cesarean Scar Pregnancy Treatment: Study Protocol for a Randomized Clinical Trial

**DOI:** 10.3389/fendo.2021.651273

**Published:** 2021-06-07

**Authors:** Yunhui Tang, Yi Zhang, Hanqing Tang, Jiahui Che, Hua Feng, Xiaoying Yao, Qi Chen

**Affiliations:** ^1^ Department of Family Planning, The Hospital of Obstetrics & Gynaecology, Fudan University, Shanghai, China; ^2^ Department of Obstetrics & Gynaecology, The University of Auckland, Auckland, New Zealand; ^3^ School of Medicine, Nantong University, Nantong, China; ^4^ Department of Gynaecology, The Hospital of Obstetrics & Gynaecology, Fudan University, Shanghai, China; ^5^ Unit of Cervical Disease, The Hospital of Obstetrics & Gynaecology, Fudan University, Shanghai, China

**Keywords:** randomized clinical trial, cesarean scar pregnancy, uterine artery embolization, intraoperative blood loss, quantitative risk-scoring system

## Abstract

**Introduction:**

Cesarean scar pregnancy affects 6% of all ectopic pregnancies in women with prior cesarean section, and there is currently no consensus on the optimal treatment. Options of surgical treatment have a risk of intraoperative blood loss; therefore, uterine artery embolization (UAE) has been considered as an option of reducing intraoperative blood loss. However, UAE may be overused in clinical practice, especially in China. We present this protocol for a randomized clinical trial investigating the necessity of performing UAE for cesarean scar pregnancy, in combination with surgical suction curettage, taking into account the different subtypes of cesarean scar pregnancy. We recently developed a risk-scoring system (QRS) to estimate intraoperative blood loss, with 93.8% sensitivity and 6.3% false negative. Through this randomized clinical trial, we will retrospectively validate the QRS score on predicting intraoperative blood loss.

**Methods and Analysis:**

We propose undertaking a randomized clinical trial sequentially recruiting 200 patients. All the patients will randomly receive ultrasound guided curettage with or without UAE. Data on the subtypes of cesarean scar pregnancy (Types 1 and II and III) detected by ultrasound will be collected before operation. The score on estimating intraoperative blood loss assessed by our recently developed quantitative risk-scoring system (QRS) will be collected before the operation. We will primarily compare the duration of the operation, intraoperative blood loss, and complications between the two groups. We will also retrospectively analyze the association of subtypes of cesarean scar pregnancy and the options of treatment and validate the QRS score. Outcomes of subsequent pregnancy within the 2-year follow-up will be secondary outcomes.

**Trial Registration Number:**

[website], identifier ChiCTR2100041654.

## Introduction

Cesarean scar pregnancy is a rare type of ectopic pregnancy implanted in the myometrium at the site of a previous cesarean section scar. Although the estimated incidence is 0.04 to 0.05% of pregnancies worldwide (1), the number of reported cases has significantly increased in the last decade. Given the risk of this life-threatening complication, management of cesarean scar pregnancy is becoming another challenge for gynecologists.

There is currently no agreement on the most optimal management of cesarean scar pregnancy. Lack of clinical trial studies with a large enough sample size to compare the advantages among the treatment options (2, 3) results in the majority of studies on cesarean scar pregnancy being reported in literature as case series (4). This consequently results in that the optimal treatment is likely based on the individual case (4, 5) and the hospital’s individual protocol or gynecologists’ experiences. Although medical treatment with systemic or local methotrexate (MTX) is popular in western countries because of the lower risk for blood loss, nearly 50% of cases diagnosed in the first trimester of pregnancy often receive surgical treatment (1). Surgical treatment includes transvaginal or hysteroscopic or laparoscopic resection and uterine artery embolization (UAE) combined with or without hysteroscopic resection or curettage (4, 6, 7).

Increasing evidence indicated that cesarean scar pregnancy is a precursor of abnormally adherent placenta later in pregnancy (8), suggesting an increased risk of a severe hemorrhage or hysterectomy could occur at the time of operation/delivery (1, 8). Although UAE can reduce the risk of intraoperative blood loss and shorten the time for *β*-hCG (human chorionic gonadotropin) normalization and hospital stay and is a safe option for caesarean scar pregnancy treatment (9, 10), a recent retrospective study reported that the necessity of UAE is low (11), suggesting that UAE may be overused in clinical practice, in particular in China (4). A recent retrospective study also reported that simple surgical suction curettage under ultrasound guidance for the treatment of cesarean scar pregnancy in a lower uterine segment is effective and safe with less intraoperative blood loss (12).

Pre-assessment of blood loss is very important to gynecologists. Our recent study reported that gestational age of cesarean scar pregnancy and blood flow around the site of the gestational sac detected by ultrasound are risk factors for potential intraoperative blood loss (13). We then developed a risk-scoring system (**Table 1**) to estimate intraoperative blood loss, and we found that women with a total score higher than three have a risk for intraoperative blood loss (13). A previous study also used a color Doppler scoring system to estimate intraoperative blood loss, according to vascularization of the pregnancy, and it indicated that women with a higher score have a higher risk of intraoperative blood loss (12).

**Table 1:** Risk-scoring system for the prediction of compression hemostasis requirement.

Variable	Score
Vascularity around the gestational sac*	
No obvious blood flow	0
Sparse or sporadic blood flow	1
Vessel looks like short-columnar pattern	2
Vessel looks cord-like pattern	3
Massive blood flow or arteriovenous fistula	4
Pregnancy duration	
≤40 days	0
41–50 days	1
>50 days	2

*As demonstrated on a color Doppler ultrasonography.

There is currently no randomized clinical trial to compare the intraoperative blood loss and postoperative outcomes by surgical treatment such as ultrasound guided curettage with or without UAE in women with cesarean scar pregnancy. Therefore, we will run a randomized clinical trial to primarily investigate whether UAE is overused in controlling intraoperative blood loss in clinical practice. We recently established a risk-scoring system (QRS) to estimate intraoperative blood loss (13), and we will then retrospectively validate the role of QRS in estimating intraoperative blood loss in this randomized clinical trial with a relatively large sample size. Recently a number of studies reported potential complications in a subsequent pregnancy in women who received surgical treatment (14). Whether the complications in a subsequent pregnancy are related to the options of the previous treatment is unknown. Therefore, the secondary measurement of this randomized clinical trial is to follow up the outcomes in a subsequent pregnancy in women treated by ultrasound guided curettage with or without UAE.

## Methods

### Study Design and Ethics

The randomized clinical trial will be conducted in a single university hospital specialized in Obstetrics and Gynaecology in China. Our tertiary hospital is the largest Women’s hospital in China. We have approximately 150 cases a year. This randomized clinical trial has been approved by The Hospital of Obstetrics & Gynaecology, Fudan University of China (Reference number KYY2020-185, and Trial registration number: ChiCTR2100041654).

### Participants

#### Sample Size Calculation

The sample size calculation was based on the reduction of the proportion of cases who lost more than 300 ml of blood during operation. Our recent study (15) showed intraoperative blood loss with a mean of 184 ml and a standard deviation (SD) of 313 ml. Converting to a Z-score, about 35% of cases would have more than 300 ml of intraoperative blood loss. With 90% power at the 5% significance level, recruiting 200 cases in total, we will be able to detect a difference of 20% between two groups.

Therefore, in this randomized clinical trial, 200 women newly diagnosed with cesarean scar pregnancy will be recruited within three years and equally divided into two groups.

### Inclusive Criteria

1. All the patients must be at least over 18 years.
2. All the patients are diagnosed with cesarean scar pregnancy in the first trimester of gestation by ultrasound followed by the guidelines.
3. The conditions of cesarean scar pregnancy in women are stable without severe vaginal bleeding, and all the patients do not need emergency surgery.
4. All the patients want to terminate their pregnancy.
5. Given written informed consent.

### Exclusive Criteria

1. Patients with other complications of gynecological diseases, such as gynecological cancers, endometriosis, and uterine myoma.
2. Patients who need emergency surgery due to cesarean scar pregnancy.
3. Patients who are not suitable for ultrasound guided suction curettage.
4. Patients who do not want to receive surgical treatment.

### Participant Enrollment

This randomized clinical trial will be introduced to potential trial participants identified from our routine clinic in our hospital by the principal or associate investigators. All the adverse events will be explained by a senior gynecologist (more than 8 years of experiences) or principal investigator or associate investigator before trial enrollment. In addition, either the principal investigator or associate investigators will be available to be contacted at all times after treatment in case of an adverse event. All the patients will be sequentially enrolled.

### Intervention

**Figure 1:**
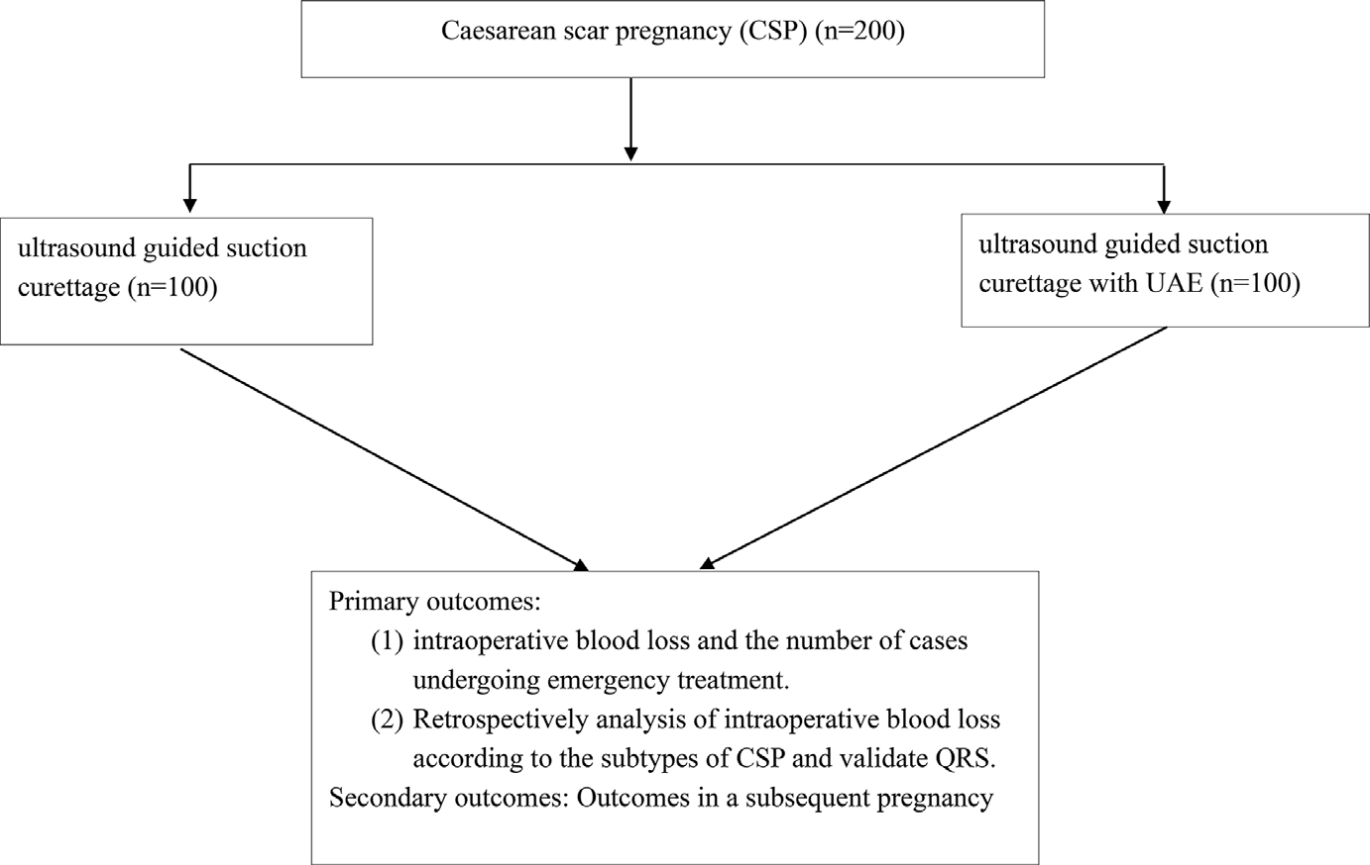
Flow chart.

Eligible patients will randomly receive ultrasound guided suction curettage with or without pre-operative UAE treatment (**Figure 1**). If there is an emergency such as severe bleeding or uterine rupture during treatment, the patients will receive hysteroscopic and/or laparoscopic resection.

### Data Collection and Follow-Up

#### Pre-Operation

Data on maternal age, parity, gravida, gestational sac age at diagnosis, the size of sac, and the number of previous cesarean section (including the indications of cesarean section) will be collected. The subtypes of cesarean scar pregnancy (Types 1 and II and III) detected by ultrasound findings following a guideline (16) will be classified. In addition, the QRS score for predicting blood loss will be also calculated based on our recently developed quantitative risk-scoring system (13).

#### Intraoperative

Duration of operation, intraoperative blood loss and intraoperative complications including emergency treatment due to the failure of first line options and modifications of treatment will be recorded. All the tissues after the operation will be sent to the department of pathology to exclude hydatidiform mole.

#### 6-Month Follow-Up Visit

Recovery of the endometrium (menstrual cycle) will be monitored by ultrasound on a routine basis. For patients who receive the treatment with UAE, liver and kidney functions will be tested one week after the operation. Cesarean scar niche will also be examined by ultrasound at 3 and 6 months.

#### 2-Year Follow-Up

Subsequent pregnancy, subsequent fertility, outcomes of subsequent pregnancy including recurrent cesarean scar pregnancy and live births will be recorded.

### Primary Outcomes

1. Comparisons of duration of operation, intraoperative blood loss and intraoperative complications between treatments by ultrasound guided suction curettage with or without UAE.
2. The numbers of cases that receive emergency hysteroscopic and/or laparoscopic resection.
3. Retrospectively analyze the intraoperative blood loss, taking into account the subtypes of cesarean scar pregnancy.
4. Retrospectively validate the QRS score to predict intraoperative blood loss.

### Secondary Outcomes

1. Subsequent pregnancy within 2 years.
2. Recurrent cesarean scar pregnancy in a subsequent pregnancy.
3. Complications in a subsequent pregnancy.
4. Subsequent infertility including cesarean scar niche.
5. Association of above outcomes and the treatment option.

### Trial Discontinuation

All the patients will be withdrawn from the trial upon participant’s request at any time point and there will be no allowance for participating in this trial.

### Statistical Analysis

All the data will be expressed as mean and SD. Student t-test or ANOVA will be performed when appropriate using Prism software version 8.4. Two side p value of less than 0.05 will be considered statistically significant.

## Discussion

Interventions for cesarean scar pregnancy could cause a massive intraoperative blood loss due to the highly vascular nature of the site of pregnancy. This consequently results in using UAE as one of the options to prevent intraoperative blood loss in clinical practice. However, a recent retrospective study suggested that UAE may be overused in clinical practice (11). Therefore, in this randomized clinical trial, we primarily aim to evaluate the necessity of UAE as a treatment option for cesarean scar pregnancy in order to control the intraoperative blood loss. We will then retrospectively take into account the subtypes of cesarean scar pregnancy to understand whether the optimal options are dependent on the subtypes of cesarean scar pregnancy.

Pre-assessment on predicting intraoperative blood loss which may be dependent on the size and/or site of the sac and the gestational age will help gynecologists select an optimal option for cesarean scar pregnancy treatment. A recent retrospective study used crossover sign (COS) to predict blood loss in surgical treatment (17), suggesting that the crossover sign (COS) is an independent risk factor for severe blood loss during treatment of cesarean scar pregnancy (18). We also recently developed a risk-scoring system (QRS) to estimate blood loss during surgical treatment with a 93.8% sensitivity and 6.3% false negative (13), which has some similarities with the COS system. Due to the relatively small sample size in our previous study, therefore, in this randomized clinical trial with a relatively large sample size, we will retrospectively validate the role of the QRS score in estimating intraoperative blood loss. We hope that this study will generate sufficient evidence for optimal cesarean scar pregnancy treatment. If effective, we believe the QRS score can be a cost-effective and safe tool to estimate intraoperative blood loss in order to reduce the unnecessary use of UAE in clinical practice.

In addition, whether recurrent cesarean scar pregnancy and the outcomes in a subsequent pregnancy including complications,and subsequent fertility in women treated for cesarean scar pregnancy are associated with their previous treatment has not been described well in literature. We hope that the secondary outcomes of the study can provide some useful information on the outcomes of a subsequent pregnancy.

## Ethics Statement

The studies involving human participants were reviewed and approved by the Hospital of Obstetrics & Gynaecology, Fudan University of China (Reference number KYY2020-185). The patients/participants provided their written informed consent to participate in this study.

## Author Contributions

YT, YZ, HT, JC, HF, XY, and QC were involved in the study design and editing and approval of the final manuscript. YZ was involved in ethical approval and clinical trial registration. All authors contributed to the article and approved the submitted version.

## Funding

This study is supported by the Medical Innovations Project of Shanghai Science and Technology Committee of China (20Y11907400).

## Conflict of Interest

The authors declare that the research was conducted in the absence of any commercial or financial relationships that could be construed as a potential conflict of interest.
